# Redox Speciation of Vanadium in Estuarine Waters Using Improved Methodology Based on Anion Exchange Chromatography Coupled to HR ICP-MS System

**DOI:** 10.3390/molecules26092436

**Published:** 2021-04-22

**Authors:** Lucija Knežević, Dario Omanović, Niko Bačić, Jelena Mandić, Elvira Bura-Nakić

**Affiliations:** 1 Division for Marine and Environmental Research, Ruđer Bošković Institute, Bijenička cesta 54, 10000 Zagreb, Croatia; lknezev@irb.hr (L.K.); omanovic@irb.hr (D.O.); Niko.Bacic@irb.hr (N.B.); 2 Laboratory for Chemical Oceanography and Sedimentology of the Sea, Institute of Oceanography and Fisheries, Šetalište Ivana Meštrovića 63, 21000 Split, Croatia; mandic@izor.hr

**Keywords:** vanadium(V) redox speciation, ion chromatography, on-column complexation, Krka River estuary, high-salinity matrix

## Abstract

An improved methodology was developed for V redox speciation in estuarine waters using a hyphenated technique consisting of ion chromatograph (IC) with an anion exchange column and a high-resolution inductively coupled plasma mass spectrometer (HR ICP-MS). This approach enables the direct determination of V(V), whereas reduced species (mainly V(IV)) are calculated by subtracting V(V) concentrations from the measured total V concentration. Based on the “on-column” V(V) chelation mechanism by EDTA, with the eluent composed of 40 mmol L^−1^ ammonium bicarbonate, 40 mmol L^−1^ ammonium sulphate, 8 mmol L^−1^ ethylenediaminetetraacetic acid and 3% acetonitrile, the method was successfully used for analyses of V redox speciation in samples taken in the vertical salinity gradient of the highly stratified Krka River estuary. Due to the matrix effects causing different sensitivities, a standard addition method was used for V(V) quantification purposes. The limit of detection (LOD) was also found to be matrix related: 101.68 ng L^−1^ in the seawater and 30.56 µg L^−1^ in the freshwater. Performed stability tests showed that V redox speciation is preserved at least 7 days in un-treated samples, possibly due to the stabilization of V-reduced species with natural organic matter (NOM). The dominant V form in the analysed samples was V(V) with the reduced V(IV) accounting for up to 26% of the total dissolved pool. The concentration of V(IV) was found to correlate negatively with the oxygen concentration. Significant removal of dissolved V was detected in oxygen depleted zones possibly related to the particle scavenging.

## 1. Introduction

Vanadium (V) is a redox-sensitive trace metal, which occurs in three oxidation states (+III, +IV and +V) in the environment [1]. Given the high concentrations in igneous, sedimentary rocks and minerals (average crustal concentrations over 200 µg g^−1^), V is also the second most abundant transition metal in seawater (around 35 nmol L^−1^) [1,2]. In freshwater, dissolved V concentrations vary between 8.5 and 22.6 nmol L^−1^, showing high dependence on the type of source rock and weathering type [3]. While vanadium in open ocean waters shows relatively conservative distribution, non-conservative behaviour is reported in coastal waters [4,5]. Due to the different chemical behaviour and toxicity depending on which species V takes the form of, speciation analysis is highly needed in order to evaluate the bioavailability and geochemical cycling of V in the environment [6]. The distribution of V redox species in natural waters is controlled by the pH, V concentration, redox potential, ionic strength of the system, chemical composition of natural organic matter and biological activity [1,2]. Thermodynamic calculations predict V(V) as a dominant species in well-oxidized marine environments, while V(IV) is stable in moderately reducing environments [1]. Under the reducing conditions, VO(OH)_3_^−^ largely dominates V(IV) speciation (with the minor share of dimmer V(IV) species-(VO)_2_(OH)_5_^−^), while in well-oxidizing environments, V(V) exists predominantly as a vanadate oxyanion (H_2_ VO_4_^−^, HVO_4_^2−^) [1,7]. However, V(IV) has been previously reported to be present even in surface oxic waters due to its ability to form stable complexes with organic or inorganic ligands in natural waters [4,8,9].

Estuaries represent an ideal natural media for studying geochemical cycling of trace metals [10,11,12]. Due to the presence of various physical and chemical gradients as well as increased primary production (in comparison to open ocean), estuaries are a source of valuable information on how these factors can affect the distribution of vanadium species and its behaviour [10,13]. Although the weathering of silicate and other minerals is thought to be a dominant factor in controlling dissolved vanadium concentration in estuaries, other secondary factors have to be taken into account as well: adsorption effects, the nature of weathering processes, redox reactions, organic complexation and various anthropogenic inputs [14]. The biogeochemical cycle of V has recently been largely influenced by anthropogenic activity [2]. Xavier et al. (2020) established moderate contamination for V in the sediment samples of Cuñaní Estuary [15]. Furthermore, Ribeiro et al. (2018) determined V to be present as the 7th most abundant trace metal in Douro River estuary and established strong contamination by the anthropogenic activity [16]. Vanadium speciation in the Krka River estuary has been recently studied in sediment samples. The stated study showed that, although V is characterized by higher background levels, the anthropogenic input of V is generally considered low for sampled sites. The speciation in bioavailable sediment fraction shows the predominance of V(IV) species [17].

The three main difficulties identified in the redox speciation of V in marine waters are (i) the preservation of the original speciation, (ii) separation and individual identification of redox specie(s) and (iii) measurement interferences in a high matrix solution. Firstly, an issue of adequate preservation/storage of the sample has to be solved with a goal of avoiding changes of the original species distribution. Different procedures were proposed, which unfortunately are not adequate for all V redox species. While the acidification of the sample is recommended for V(V), the addition of chelating agent (EDTA) was used to maintain V(IV) stability [18,19,20,21]. A further step in V speciation analysis includes the choice of the V species separation method, mainly consisting of pre-concentration procedures due to the low concentration and complex environmental matrices [22,23,24]. Such techniques (chelation and extraction; precipitation and pre-concentration on ion exchange resins) often require large sample volumes due to their low detection limit and imply the usage of complexation ligands [21,25,26,27]. The off-line application of these methods is often under a high risk of sample contamination, while an on-line setup significantly increases analysis costs [23,24]. Most of the other on-line techniques (flow injection analysis; spectrophotometry coupled to devices of higher detection limit) require additional treatment of the samples, which is not desirable (addition of complexing ligands; lowering natural pH of the samples) due to the high risk of sample contamination or change in the present species [23]. Due to their simple and fast measurement, on-line separation chromatographic techniques (ion chromatography, IC; liquid chromatography, LC) are often hyphenated with analytical instrumentation of the lower detection limit, such as inductively coupled plasma-optical emission (ICP-OES) or inductively coupled plasma mass spectrometry (ICP-MS) [22,28]. However, ICP-MS is more often used for vanadium determination, mainly because it enables element selective measurement with higher sensitivity, appropriate for the measurement of natural samples [23]. On the other hand, this analytical setup can be prone to matrix effects originating from environmental samples, especially those of high salinity [28,29,30,31]. Interactions of matrix ions with column, as well as interferences originating from spectrometric devices, can lead to peak broadening, suppression or enhancement of the analyte signal, co-elution effects, microgradient elution, etc. [23,28].

The published method for V speciation in the coke pore water samples and bacterial cultures by Li and Le using the HPLC-ICP-MS system on an anion exchange column served as a basis for V redox speciation in the samples of the Krka River estuary [18]. However, processed samples in the work of Li and Le were of very low salinity in comparison to the varying salinity samples of the Krka river estuary. To our knowledge, only one study using ion-pair reversed LC deals with the V redox speciation in high-salinity oceanic waters where the samples were pre-treated by the addition of EDTA prior to IC separation (diluted two-fold with the mobile phase) [32]. Consequently, the aims of this work are as follows: (i) improve analytical determination of vanadium species using IC on anion exchange column coupled HR ICP-MS taking into account matrix effects originating from estuarine samples of variable salinity; (ii) address species preservation and sample pre-treatment in order to avoid changes in original speciation; and (iii) application of the developed method for the determination of vanadium redox speciation in estuarine waters (the Krka River estuary, Croatia).

## 2. Results and Discussion

### 2.1. Methodology Improvements

#### 2.1.1. Effect of Sample Matrix

**Figure 1:**
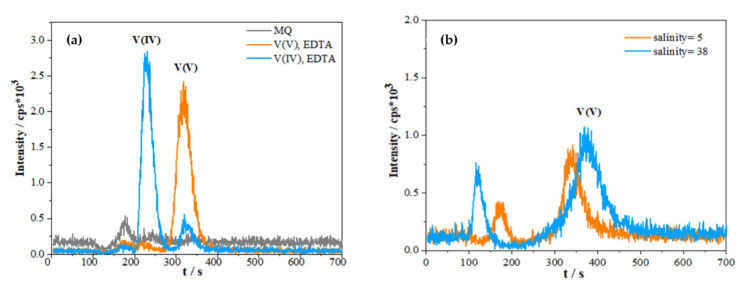
(**a**) Chromatograms of solutions containing 40 nmol L^−1^ V(V) (orange line) and 40 nmol L^–1^ V(IV) (blue line) in MQ water with the EDTA added in solution (3 mmol L^−1^, pH = 7) compared to blank (MQ water, grey line) using IC–ICP–MS. (**b**) Comparison of chromatograms obtained by IC-HR ICP-MS at different salinities; total V and V(V) concentration measured in samples collected at “Vrnaža port” (VP) sampling station (orange line: environmental sample of salinity = 5, V_tot_ = 24.5 ± 1.4 nmol L^−1^, V(V) = 21.8 ± 0.1 nmol L^−1^; blue line: environmental sample of salinity = 38, V_tot_ = 37.0 ± 0.8 nmol L^−1^, V(V) = 31.1 ± 3.34 nmol L^−1^). All chromatograms (**a**,**b**) were measured using the following eluent composition: 40 mmol L^−1^ HCO_3_^–^, 40 mmol L^–1^ SO_4_^2–^, 8 mmol L^–1^ EDTA and 3% acetonitrile.

Speciation analysis of V species is achieved by their complexation with EDTA, which enables the simple separation of the species in the form of corresponding V-EDTA negatively charged complexes using anion-exchange-based IC [33]. Figure 1a shows IC-HR-ICP-MS chromatograms of V(IV)-EDTA and V(V)-EDTA complexes prepared in Milli-Q water. The obtained elution times for two species are in agreement with previous reports [18,33,34]. While the IC-separation of V species in Milli-Q water provides the two peaks at different elution times, the chromatograms obtained in natural waters of different salinities are more complicated (Figure 1b). In both saline samples, the two well-separated peaks appeared, the first peak between 100 and 200 s and the second peak between 300 and 400 s. While the second peak corresponds to the elution of V(V), the first one is not related exclusively to V(IV), but is ascribed to the partial elution of V-species in the “void-volume” (this peak is hereafter termed as the “pre-peak”).

The appearance of a “pre-peak” in void volume using an anion-exchange column is a known issue in samples with high concentration of anions, such as marine waters [35,36,37,38]. In addition, the peak of V(V) and the peak within the void volume shift depending on the ionic strength of the sample. The influence of the matrix composition (salinity) is clearly visible in two chromatograms in Figure 1b. It is likely that the high concentration of chloride anions originating from the samples interferes with the equilibria on the anion exchange column, since their concentration far exceeds the analyte concentration. Chloride anions are retained on the separation column and partially saturate the active sites of the column. Consequently, not all V species present in the sample successfully adsorb on the column, which leads to their partial elution within the void volume. The amount of vanadium successfully adsorbed on the anion exchange column depends on the concentration of interfering anion(s) [31]. Although in the investigated “Vrnaža port” (VP) samples, an appreciable amount of V(IV) was determined (see discussion later), the chromatographic peaks corresponding to V(IV) were not observed. As seen in Figure 1a, V(IV) elutes closer to the void volume in comparison to V(V). Possibly, the low concentration of the present V(IV), together with the high ionic strength of the investigated samples, causes the complete elution of V(IV) within the void volume. It should be highlighted that the “pre-peak” in the chromatograms in Figure 1b originates from the V, and not spectral interference caused by the formed ClO^+^, since the medium resolution used for the measurement is sufficient to fully eliminate the spectral interference [35].

By comparing the chromatograms present in Figure 1b, an increase in the “pre-peak” in the measured samples of higher salinity and V concentration can be observed. However, despite the higher total V concentration (V_tot_) in the sample of higher salinity, the peak attributed to V(V) is almost of the same size as the one at lower salinity. This is due to the lower efficiency of the V-EDTA species being retained in the column due to the interfering anion(s) and the lower sensitivity of HR ICP-MS caused by the ionization suppression of plasma in the heavier matrix [39]. Described behavior is the main reason for implementation of standard addition method for V(V) quantification instead of external calibration.

#### 2.1.2. Species Preservation and Sample Pre-Treatment

**Figure 2:**
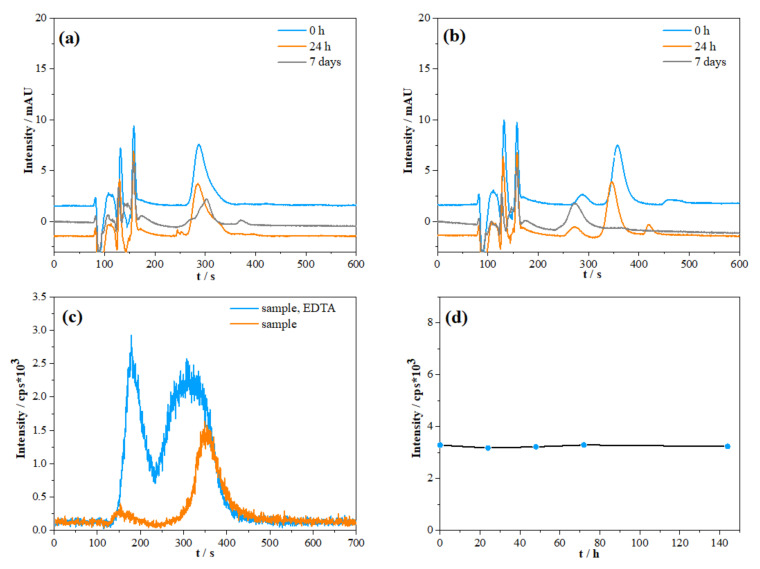
Temporal evolution of IC-UV/VIS chromatograms of V(V) (1 µmol L^–1^) and EDTA (3 mmol L^−1^) in two solutions of different pH values: (**a**) pH of 7 and (**b**) 2 (blue curve: solution measured right after preparation; orange curve: solution measured after 24 h; grey curve: solution measured after 7 days). (**c**) Comparison of different approaches to sample storage measured using IC–ICP–MS (orange curve: chromatogram of a sample filtered on-site and stored at +4 °C; blue curve: chromatogram of a same sample containing ligand (3 mmol L^–1^ EDTA) added on-site, after filtration. (**d**) Stability of V(V) (40 nmol L^−1^) spiked in natural sample of the Krka River estuary during the time period of 144 h.

The preservation of samples with the goal of V speciation studies is often based on the addition of a strong chelator (mostly EDTA) on-site and/or prior to analysis in order to stabilise reduced species (if present) against oxidation [18,20]. In order to identify if any changes in vanadium species occur in the sample solution after EDTA addition, a reported sample handling approach was tested in model solutions prior to the analysis of environmental samples [18,20,32,40]. Since predominant species in oxic water samples are expected to be mostly vanadate ions (V(V)), tested model solutions were prepared by using the V(V) standard [1,2]. Stability tests of vanadium species complexed with added EDTA in model solutions at different pH values are shown in Figure 2a,b (UV/Vis detector was used for measurement). It is apparent that the V(V)-EDTA complex shows significant instability at the two different pH values. Under both neutral and acidic conditions, a decrease in peak intensity for V(V) with the time was observed. Measured solutions at pH = 2 showed a reduction in V(V) in V(IV) after a 7-day period. On the other hand, chromatograms of solutions at pH = 7 showed slight changes in peak position after just 24 h, which could possibly be attributed to the slight increase in the pH of the solutions within the measurement time frame [32]. Under acidic conditions, the peak at 280 s elution time, which can be attributed solely to V(IV)-EDTA produced in the reduction processes between V(V) species and EDTA, showed a further increase. A higher reduction rate on lower pH values, in comparison with more alkaline pH, can be attributed to the more pronounced V(IV) stability against oxidation at the lower pH values. V(V) has been already shown to gradually convert to the V(IV) species in the presence of EDTA [41,42,43,44].

The same preservation approach was tested on natural samples of the Krka River estuary (Martinska (MA), sampling station). Figure 2c shows a comparison of chromatograms from differently stored (but otherwise the same) samples with the on-site addition of EDTA and without EDTA. It is obvious that sample storage using EDTA for the preservation of vanadium redox species is not adequate. The obtained chromatogram with EDTA addition, where severe peak broadening and an increase in “pre-peak” (peak at about 180 s) were observed, imply that a change in the original vanadium speciation is likely to occur. Contrarily, a well-shaped peak of V(V) was obtained in samples without EDTA addition. Furthermore, a prolonged stability test over a 6-day period with spiked estuarine samples (Figure 2d) revealed that V speciation under natural conditions was successfully preserved. Based on the conducted stability tests, it was decided that on-site filtration and storage of the samples at +4 °C would be the optimal sample handling strategy.

Following the mentioned non-preservation approach, an estuarine sample (Skradin Bridge (SB) site: with total dissolved V of 25.3 ± 1.0 nmol L^−1^) was analysed twice within a period of 6 days: the first measurement performed after the sampling gave a V(V) concentration of 24.2 ± 0.5 nmol L^−1^, whereas 22.9 ± 1.0 nmol L^−1^ was found in the same sample after 6 days. Assuming that the obtained difference in V(V) concentrations between these two measurements was withing the usual experimental error (<10%), it could be concluded that the redox speciation of V in an untreated sample over the period of at least 6 days is preserved. Our stability results are consistent with the results of other authors [45,46,47], suggesting the stabilization of V(IV) in oxic conditions by complexation with organic and/or inorganic ligands in natural samples [2,3,4,19,45]. It is thus presumed that the chosen sample storage (without any chemical preservation) would not affect the speciation of vanadium species in the sample, even if reduced species are present.

#### 2.1.3. Optimisation of Eluent Composition

**Figure 3:**
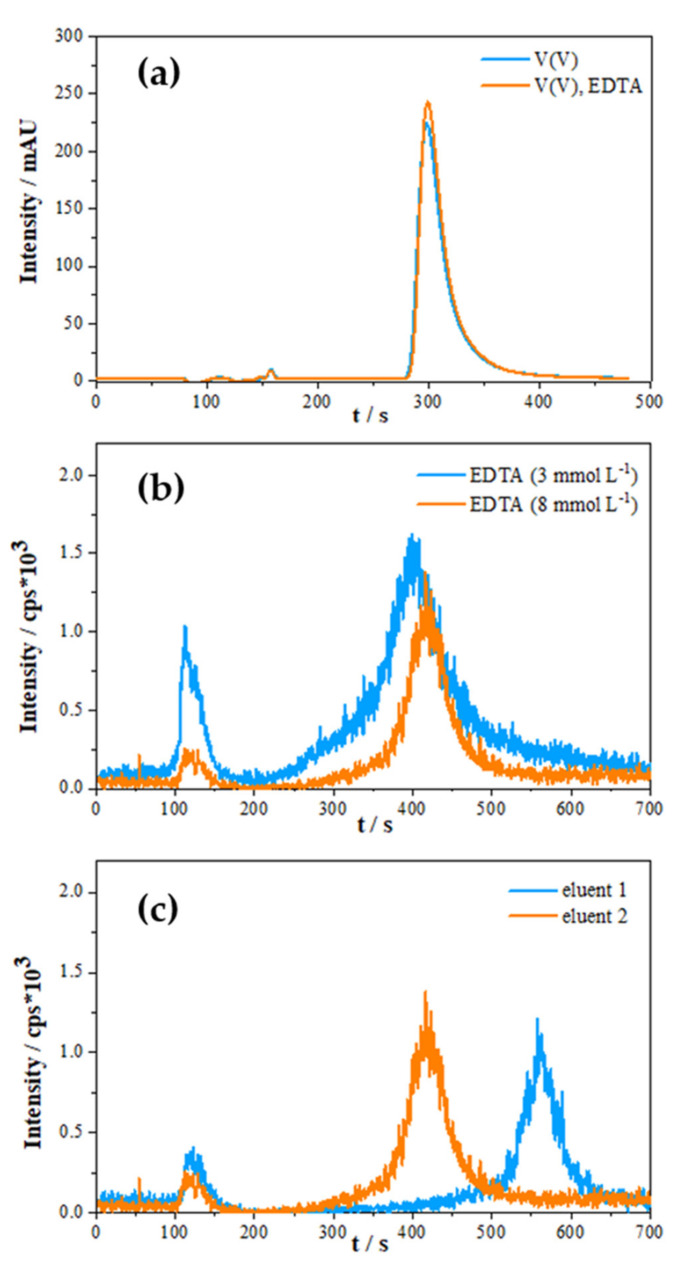
(**a**) Comparison of chromatograms for measured solutions using eluent composed from 40 mmol L^–1^ HCO_3_^–^, 40 mmol L^–1^ SO_4_^2–^, 8 mmol L^–1^ EDTA and 3% acetonitrile on IC-UV/Vis containing: V(V) (0.1 mmol L^−1^) in MQ–blue line; V(V) (0.1 mmol L^−1^) complexed withEDTA (1 mmol L^−1^) in MQ– orange line; (**b**) comparison of IC–ICP–MS chromatograms in seawater sample (salinity= 38). Eluent containing 3 mmol L^–1^ EDTA, 40 mmol L^–1^ HCO_3_^–^, 40 mmol L^–1^ SO_4_^2–^ and 3% acetonitrile (blue line) and 8 mmol L^–1^ EDTA, 40 mmol L^–1^ HCO_3_^–^, 40 mmol L^–1^ SO_4_^2–^ and 3% acetonitrile (orange line) are used. (**c**) Eluents containing 80 mmol L^–1^ HCO_3_^−^, 8 mmol L^–1^ EDTA and 3% acetonitrile (blue line, eluent 1) or 40 mmol L^–1^ HCO_3_^–^, 40 mmol L^–1^ SO_4_^2–^, 8 mmol L^–1^ EDTA and 3% acetonitrile (orange line, eluent 2) are used.

An eluent consisting of 80 mmol L^–1^ NH_4_ HCO_3_, 2 mmol L^–1^ EDTA and 3% acetonitrile was suggested by Li and Le as optimal composition on valid separation of vanadium species and was used here as a starting composition for further optimisation [18]. As explained in the previous section, the addition of EDTA in the samples is not desirable due to the instability of the V(V)-EDTA. Several authors used EDTA in eluent solution to enhance the peak stability and to induce complexation of the vanadium species on the pre-column or on-column [40,48,49]. It was found that the on-column complexation with EDTA added only in the eluent would be sufficient and fast enough to separate and quantify vanadium species. The on-column formation of V(IV)-EDTA and V(V)-EDTA is enabled due to the high formation constants of V species with EDTA (*K*_f_[V(IV)-EDTA = 18.80; *K*_f_[V(V)-EDTA = 15.55) [48]. Chromatograms obtained with the model sample containing solely V(V) or both V(V) and EDTA, measured on IC-UV/Vis, are presented in Figure 3a. Retention times of eluted V(V) in both solutions are the same, suggesting that complete on-column complexation occurred in the case of the solution that did not contain EDTA.

Figure 3b presents two chromatograms which demonstrate the influence of different concentrations of EDTA in eluent solution on the separation of vanadium species in the seawater sample (Salinity = 38) using the IC-ICP-MS system. Compared to the previously suggested lower EDTA concentration [18], at a higher EDTA concentration, the V(V) peak is much better resolved due to the lower under-peak background, whereas the “pre-peak” intensity (signal from the void volume) is strongly diminished. A decrease in the “pre-peak” intensity with the usage of a higher concentration of EDTA in eluent can be linked with the parallel increase in the overall ionic strength of the eluent and promotion of V(V)-EDTA complexation on the anion-exchange column. By this modification, the concentration of V(V) eluting within the void volume decreased, and a more successful separation of V(V) on the anion exchange column was accomplished. In addition, lower background intensity on chromatograms measured on eluent containing a higher EDTA concentration was beneficial for analytical purposes, which led to more reliable results. Note that an increase in EDTA on concentrations above 8 mmol L^−1^ did not give further chromatogram enhancements. Consequently, the concentration of 8 mmol L^−1^ of EDTA in eluent was chosen to be optimal for the determination of vanadium species.

During the process of elution, eluents have to be of sufficient ionic strength to allow retained anions from the sample to be eluted. In the samples of high salinity where the concentration of matrix anions far exceeds the concentration of vanadium species, eluent ions have to efficiently remove retained matrix anions from the sample and allow the on-column anion exchange of V(V)-EDTA complexes. Thus, eluent composition was further optimized by adding ammonium sulphate to the eluent in order to increase the ionic strength of the eluent. A comparison of IC-ICP-MS chromatograms obtained with eluent composition suggested by Li and Le (2007) [18] (eluent 1) and our optimized eluent solution (eluent 2) is presented in Figure 3c. The most obvious differences are in retention times and “pre-peak” intensities.

The longer retention time obtained with eluent containing solely ammonium bicarbonate was partly caused by the lower pH compared to the bicarbonate–sulphate mixture (6.0 vs. 8.5). Additionally, the peak shift can be attributed to a slightly higher ionic strength of bicarbonate–sulphate eluent, allowing elution of the species at a lower retention time, which enables shorter analysis. The bicarbonate–sulphate mixture was also found to be more efficient in reducing “pre-peak” intensity, preventing a greater influence of the sample anion effects. The newly proposed eluent composition with pH = 8.5 is also more beneficial, since the higher pH values favour the formation of V-EDTA complexes. [18,40,48,49].

Finally, a concentration of 40 mmol L^–1^ ammonium sulphate, 40 mmol L^–1^ ammonium bicarbonate, 8 mmol L^–1^ EDTA and 3% acetonitrile was selected as an optimal eluent composition, since this composition shortens the retention time of V(V), slightly increases V(V) peak intensity and decreases the void volume “pre-peak” intensity.

### 2.2. Distribution of V Redox Speciation in Krka River Estuary Samples

**Figure 4:**
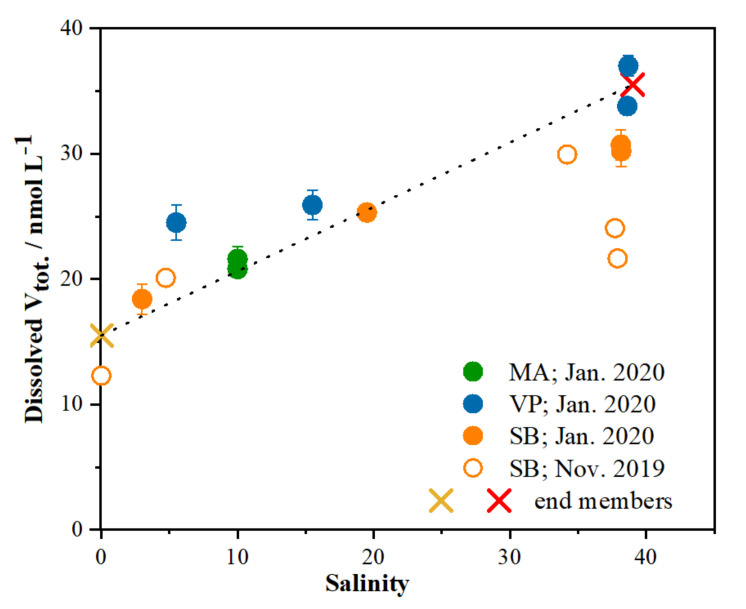
The relationship between the total dissolved vanadium concentration and salinity at the MA (green circles), VP (blue circles) and SB (full orange circles—January 2020; empty orange circles—November 2019) sites. The theoretical dilution line between the two end members, open sea (red cross) and the Krka River (light brown cross), is represented by the black line.

Respecting the optimised procedures for sampling storage and vanadium redox speciation, a selected set of estuarine samples covering the full salinity range, characterized by different physico-chemical and anthropogenic conditions, was analysed. The dependence of total vanadium concentration on the salinity for the three sampling sites is shown in Figure 4. Concentrations of total vanadium in two end members (freshwater part and open sea) are also marked and connected with a line corresponding to the theoretical dilution line. Basically, a near-conservative behaviour was obtained for vanadium in the surface brackish layer in the absence of additional V input. A small positive deviation at low salinity was ascribed to the anthropogenic influence within the harbour area (VP site), whereas negative deviation at high salinities was observed for the most upstream site in the bottom seawater layer. This estuarine segment is characterized by a higher concentration of suspended particulate matter and a long residence time of the bottom seawater. Similar processes related to the particle scavenging, as well as to biogenic adsorption and/or biological uptake, are also found for the nearby Zrmanja River estuary [11].

**Figure 5:**
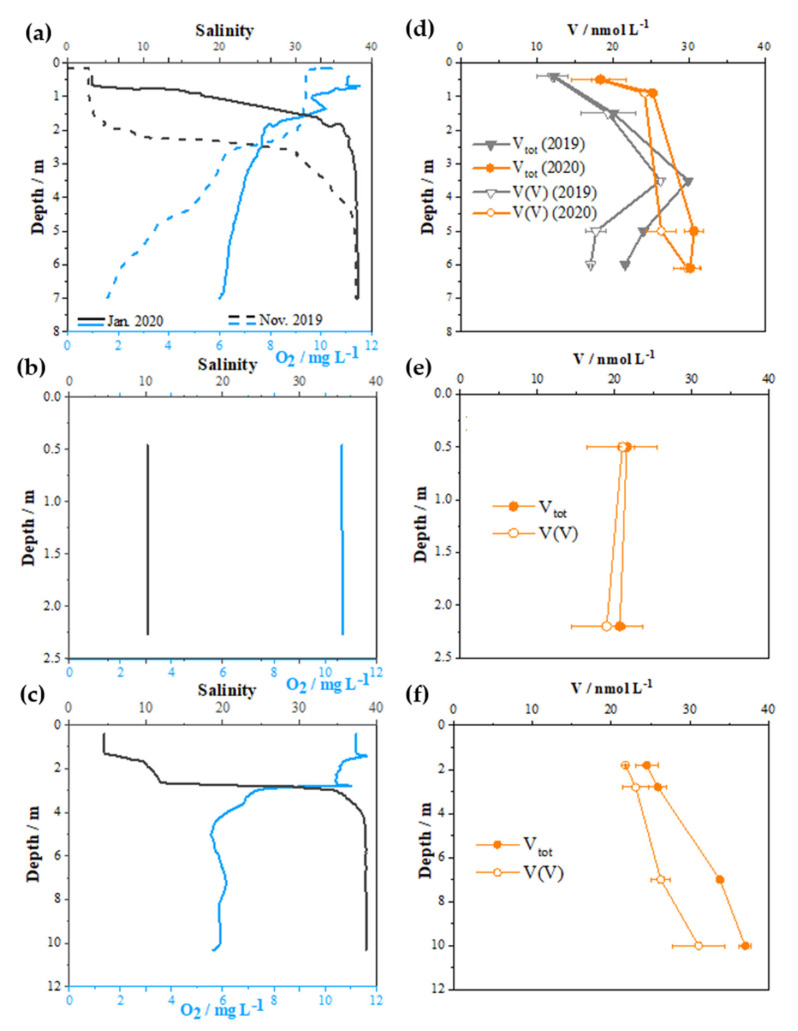
Vertical profiles of salinity and dissolved oxygen, and total dissolved V (full circles) and dissolved V(V) (empty circles) for SB (**a** and **d**), MA (**b** and **e**) and VP (**c** and **f**) sites. Dashed lines on panel (**a**) as well as grey symbols on panel (**d)** represent November 2019 sampling at the SB station, while the rest of the data represent January 2020.

**Figure 6:**
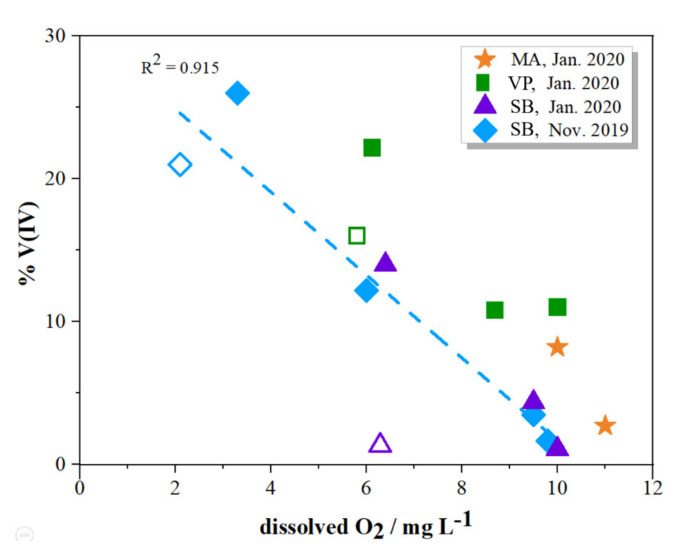
Percentage of reduced species in the water column of MA (orange markers), VP (green markers) and SB (purple markers—January 2020; blue markers—November 2019) sampling stations in relation to the dissolved oxygen concentration. Empty markers represent bottom seawater layer for each sampling station.

Vertical distributions of the total dissolved V and V(V) in the water column of the three sampling sites are presented in Figure 5, whereas numerical results are provided in Table S1. The increase in V concentration with the depth is in line with the above presented salinity dependency. As expected, the predominant V specie in all samples is V(V), accounting for more than 74% of the total V. The concentrations of reduced V(IV) species were higher in the deeper seawater layer than in the surface layer. Further examination revealed that the percentage of V(IV) was highly related to the oxygen concentration, as presented in Figure 6. Such dependence could be justified by different redox conditions and water chemistry, as detailed in Wang et al. [45].

In November, when the bottom seawater layer at SB site was highly depleted in oxygen, the concentration of the V(IV) was the highest, accounting for 26% of the total dissolved V. The development of hypoxia was found to be common in the autumn/winter period in this part of the estuary and is explained by the degradation of organic matter derived from high primary production in the summer period [12,50]. However, oxygen depletion does not seem to be the only factor affecting the distribution of the V redox species. Interestingly, concentrations of reduced species are higher on the VP sampling site compared to sampling site SB for the same concentration of dissolved oxygen. A possible contributing factor to the stability and increased share of reduced species at the VP location is linked with anthropogenic input characteristic for this part of the estuary [12,51,52].

It is interesting to note that in January 2020, at the SB sampling site, V(IV) in the bottom layer was not observed. However at the bottom layer of VP sampling site V(IV) was detected (up to ~15% of total dissolved V), although the O_2_ concentration between sampling sites differed only slightly (Figure 6, empty marker points). It is likely that the adsorption of V(IV) onto particles and colloids occurred, especially in the upstream parts of the estuary where the enrichment of particulate matter was reported [12].

In order to obtain information on the possible contributing factors of reduction mechanism of V(V) species in oxic water system, a study on the composition and the behaviour of the organic matter and particulate matter would be needed in further studies.

## 3. Materials and Methods

### 3.1. Study Site

**Figure 7:**
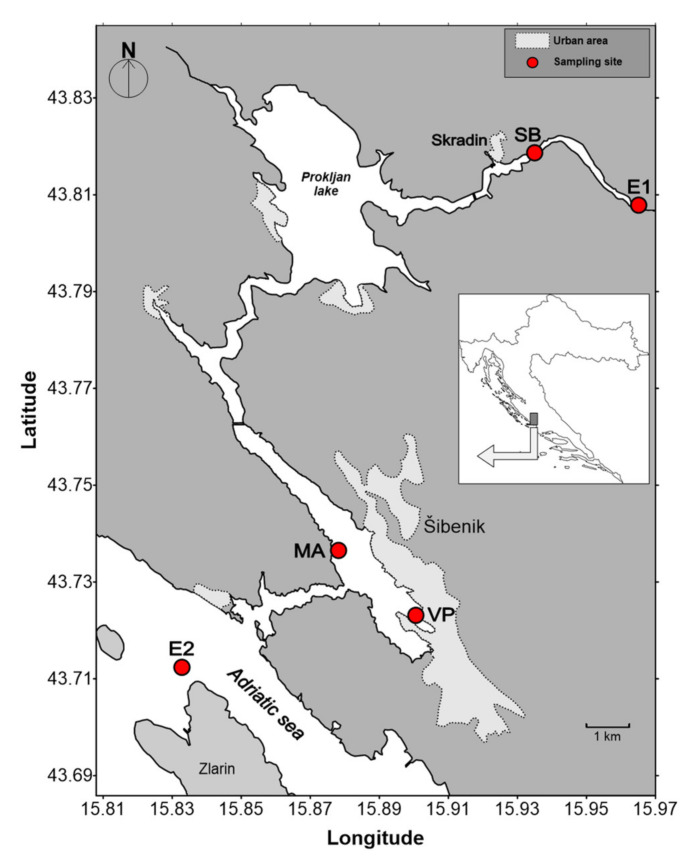
Map of the Krka River estuary with indicated sampling sites.

The Krka River is a 49 km-long river with an average flow varying between 40 and 60 m^3^ s^−1^. The estuary starts below the waterfalls of Skradinski Buk and ends at the Šibenik channel, with a total length of 23.5 km [53] (Figure 7). Due to its low tidal range of 0.2 to 0.5 m and sheltered geographical position, the Krka River estuary belongs to a highly stratified type of estuaries [54]. The surface current is directed towards the sea, while the bottom current of seawater can be followed upstream to the Skradinski Buk [54]. Vertical gradient is characterized by three layers: a surface freshwater/brackish layer, a freshwater-seawater interface and a seawater layer. Halocline is usually positioned between 1.5 and 3 m, with the thickness of the halocline layer varying between a few cm up to 1 m [12,54]. Special characteristics make this sampling location ideal for evaluating the mobility and fate of trace metals and natural organic matter [12,53,55,56,57]. Trace metals have been previously studied, although mainly in reference to their total concentrations [12,51]. In addition, some of the recent studies on sediments of the Krka River estuary showed detectable anthropogenic input (due to the nautical tourism) in local restricted areas, although the estuary generally is not considered polluted [51,52,58].

The sampling sites were selected to represent typical estuarine zones of varying anthropogenic input, physico-chemical gradients and biological activity [57,59]. Sampling was conducted on November 2019 and January of 2020 at three different locations along the Krka River estuary (Figure 7): Skradin bridge (denoted as SB), Martinska (denoted as MA) and Vrnaža port (denoted as VP), as well as two end-member locations: the Krka River water above the Skradinski Buk waterfalls (E1) and coastal seawater (E2). With the exception of two end members and the MA sampling station (where the brackish layer was sampled only), samples of the surface brackish layer, freshwater–seawater interface and seawater layer were collected at the remaining two sampling sites. Salinity of processed sampling sites varied from 4 to 38 salinity units (additionally shown in Section 2.2, Figure 4).

### 3.2. Equipment and Chemicals

For the determination of vanadium species, an ion chromatograph (Eco IC, Metrohm) with an anion exchange column (Metrosep A Supp 5–50/4.0, 50.0 mm length, 4.0 mm of inner diameter) was used. In the process of method development, two different instrumental systems for the determination of V were used: the 944 Professional UV/Vis Detector Vario and a high-resolution inductively coupled plasma mass spectrometer (HR ICP-MS, Element 2, Thermo). Both were operated at a flow rate of 0.3 mL min^-1^, controlled by the IC system. HR ICP-MS was operated at *m/z* = 50.942 mass detection since it is the dominant naturally occurring V isotope (99.76%). In order to avoid isobaric interferences (the dominant one being ^35^ Cl^16^ O^+^ due to the high salinity of the samples), ICP-MS measurements were heldat medium resolution (M/ΔM = 4000). The temperature of the anion exchange column was ambient (~22 °C, air conditioned), and the sample injection volume was 100 µL. Details on other operating conditions of the HR ICP-MS system are available in the ESI material.

Vanadium (IV) stock standard solutions of 0.02 mol L^−1^ were prepared by dissolving 0.50 g of VOSO_4_ × 5H_2_ O (VWR BDH Prolabo 132 Chemicals) in 100 mL of MQ water (18.2 MΩ cm, Milipore, USA). A stock solution of V(V) (0.02 mol L^–1^) was prepared by dissolving 0.23 g of ammonium metavanadate (VWR BDH Prolabo Chemicals) in 2 mL concentrated HNO_3_ (KEMIKA d.d.), then diluting to 100 mL with MQ water (18.2 MΩ cm, Milipore, USA). The used chemicals for eluent preparation were: acetonitrile (VWR BDH Prolabo Chemicals), ethylenediaminetetraacetic acid (EDTA) (VWR BDH Prolabo Chemicals), ammonium hydrogen carbonate (NH_4_ HCO_3_) (VWR BDH Prolabo Chemicals) and ammonium sulphate ((NH_4_)_2_ SO_4_) (VWR BDH Prolabo Chemicals). Indium standard solution for V_tot_ measurements was prepared by the dilution of 1000 mg L^−1^ AAS standard (Fluka).

Bottles for sampling and sample storage (PFA-perfluoroalkoxy, Nalgene) were previously cleaned with 10% HNO_3_ of analytical reagent grade, rinsed thoroughly with Milli-Q water (18.2 MΩ cm, Millipore, USA) and filled with Milli-Q water until use. Upon sampling, the sampling bottles were rinsed with the sample.

After collection, the final samples were filtered with previously pre-cleaned 0.22 µm pore size filters (cellulose-acetate, Minisart, Sartorius; precleaned in HNO_3_ and rinsed with Milli-Q water) and stored at natural pH at +4 °C until analysis. Vertical profiles of physical and chemical parameters (salinity, temperature, pH, dissolved oxygen and chlorophyll a) were measured in situ, using an EXO2 multiparameter CTD probe (YSI).

### 3.3. Determination of V_tot_ and V Redox Species

V_tot_ was measured using HR ICP-MS analytical instrumentation. Quantification was performed by using external matrix matching calibration. Briefly, the stock solutions with increasing V concentrations (0, 0.1, 1 and 10 µg L^−1^) for external calibration were prepared in a 10× diluted CASS-5 (nearshore seawater reference material for trace metals, NRC, Canada) certified sample. The internal In standard (10 ppb) was added to both calibration solutions and sample solutions in order to minimise the matrix effect affecting the accuracy of the V_tot_ measurement. The same certified sample was also used for the quality control (QC) and measured as every 5th sample in the sequence. The obtained V concentrations agreed within 10% with the certified value. Working standards, as well as blank solutions, were prepared with the addition of 2% high-purity HNO_3_. For total V concentration measurements, samples were diluted 10× with 2% high-purity HNO_3._

Vanadium speciation on natural samples was conducted using anion exchange ion chromatography coupled to HR ICP-MS. Filtrated samples of Krka river were directly processed on stated analytical instrumentation, and successful separation of V(V) species was achieved. Instead of calibration curves suggested by Li and Le, in our work, for the accurate quantification of V(V), we used a standard addition method (peak heights of the chromatograms were used as an analytical signal). V(IV) species quantification was achieved by the subtraction of determined V(V) species from the total V measured. The limit of detection was found to be 2 nmol L^−1^ in the seawater and 0.6 nmol L^-1^ in the freshwater, while recoveries were within 5%.

## 4. Conclusions

An improved method for vanadium redox speciation using anion exchange ionic chromatography (IC) coupled with high-resolution inductively coupled plasma mass spectrometry (HR ICP-MS) was described and applied to the environmental samples of the Krka River estuary. By modification and optimization of eluent composition (40 mmol L^−1^ HCO_3_^−^, 40 mmol L^−1^ SO_4_^2−^, 8 mmol L^−1^ EDTA and 3% acetonitrile), eluent pH values and sample pre-treatment approach V(V) species were successfully separated even in the samples of high salinity. Due to the complex matrix of natural samples, V(V) species were determined using a standard addition method, whereas reduced species (mostly V(IV)) were determined by subtracting the V(V) concentration from the total V. Preservation and storage tests showed that unlike the previously suggested EDTA addition to samples upon the sampling, the speciation was preserved for at least 7 days without any ligand additions, if samples were kept at natural pH and at 4 °C.

Conservative behaviour of V species was found in the salinity gradient of the surface estuarine layer. It was found that V(V) species were predominant redox species in all samples. However, a high share of V(IV) (up to 26%) was found in the samples taken from the oxygen-depleted water layers at the upper part of the estuary. Additionally, higher concentrations of reduced species were detected at the sampling locations linked with anthropogenic input. It was shown that the stability of reduced species is preserved even in the oxic conditions, suggesting the interaction of vanadium species with organic or inorganic ligands present in the water column of sampling stations.

## Supplementary Materials

The following are available online, Table S1. Numerical values of total dissolved V(V) concentration and calculated percentage within the Krka River estuary samples. Table S2. Operating conditions of High Resolution Inductively Coupled Plasma Mass Spectrometry (HR ICP-MS).

## References

[1] Huang J.H., Huang F., Evans L., Glasauer S. (2015). Vanadium: Global (bio)geochemistry. Chem. Geol..

[2] Gustafsson J.P. (2019). Vanadium geochemistry in the biogeosphere –speciation, solid-solution interactions, and ecotoxicity. Appl. Geochem..

[3] Bernárdez P., Ospina-Alvarez N., Caetano M., Prego R. (2013). Fluvial contributions of nutrient salts, dissolved trace elements and organic carbon to the sea by pristine temperate rivers (SW Europe). Environ. Chem..

[4] Wang D., Sañudo Wilhelmy S.A. (2009). Vanadium speciation and cycling in coastal waters. Mar. Chem..

[5] Collier R.W. (1984). Collier Particulate and dissolved vanadium in North Pacific Ocean. Nature.

[6] Pyrzyńska K. (2006). Selected problems in speciation analysis of vanadium in water samples. Chem. Anal..

[7] Costa Pessoa J. (2015). Thirty years through vanadium chemistry. J. Inorg. Biochem..

[8] Xiong C., Qin Y., Hu B. (2010). On-line separation/preconcentration of V(IV)/V(V) in environmental water samples with CTAB-modified alkyl silica microcolumn and their determination by inductively coupled plasma-optical emission spectrometry. J. Hazard. Mater..

[9] Shi Y.X., Mangal V., Guéguen C. (2016). Influence of dissolved organic matter on dissolved vanadium speciation in the Churchill River estuary (Manitoba, Canada). Chemosphere.

[10] Strady E., Blanc G., Schäfer J., Coynel A., Dabrin A. (2009). Dissolved uranium, vanadium and molybdenum behaviours during contrasting freshwater discharges in the Gironde Estuary (SW France). Estuar. Coast. Shelf Sci..

[11] Fiket Ž., Ivanić M., Turk M.F., Mikac N., Kniewald G. (2018). Distribution of trace elements in waters of the zrmanja river estuary (eastern adriatic coast, croatia). Croat. Chem. Acta.

[12] Cindrić A.M., Garnier C., Oursel B., Pižeta I., Omanović D. (2015). Evidencing the natural and anthropogenic processes controlling trace metals dynamic in a highly stratified estuary: The Krka River estuary (Adriatic, Croatia). Mar. Pollut. Bull..

[13] Chester R., Department of Earth Sciences (1990). Marine Geochemistry.

[14] Shiller A.M., Mao L. (2000). Dissolved vanadium in rivers: Effects of silicate weathering. Chem. Geol..

[15] De Arruda Xaviera D., dos Santos V.F., de Miranda A.G.O., Berrêdo J.F. (2020). Determination of background geochemistry of an Amazon estuary: The Cuñaní Estuary–Amapá. Mar. Pollut. Bull..

[16] Ribeiro C., Couto C., Ribeiro A.R., Maia A.S., Santos M., Tiritan M.E., Pinto E., Almeida A.A. (2018). Distribution and environmental assessment of trace elements contamination of water, sediments and flora from Douro River estuary, Portugal. Sci. Total Environ..

[17] Knežević L., Cukrov N., Bura-Nakić E. (2020). Ion-exchange chromatography as a tool for investigating vanadium speciation in sediments: Preliminary studies. J. Soils Sediments..

[18] Li X.S., Le X.C. (2007). Speciation of vanadium in oilsand coke and bacterial culture by high performance liquid chromatography inductively coupled plasma mass spectrometry. Anal. Chim. Acta..

[19] Hirayama K., Kageyama S., Unohara N. (1992). Mutual separation and preconcentration of vanadium(V) and vanadium(IV) in natural waters with chelating functional group immobilized silica gels followed by determination of vanadium by inductively coupled plasma atomic emission spectrometry. Analyst.

[20] Colina M., Gardiner P.H.E., Rivas Z., Troncone F. (2005). Determination of vanadium species in sediment, mussel and fish muscle tissue samples by liquid chromatography-inductively coupled plasma-mass spectrometry. Anal. Chim. Acta..

[21] Wuilloud R.G., Wuilloud J.C., Olsina R.A., Martinez L.D. (2001). Speciation and preconcentration of vanadium(V) and vanadium(IV) in water samples by flow injection-inductively coupled plasma optical emission spectrometry and ultrasonic nebulization. Analyst.

[22] Pyrzyńska K., Wierzbicki T. (2004). Determination of vanadium species in environmental samples. Talanta.

[23] Chen Z.L., Owens G. (2008). Trends in speciation analysis of vanadium in environmental samples and biological fluids—A review. Anal. Chim. Acta..

[24] Cornelis R., Caruso J.A., Crews H., Heumann K.G. (2005). Handbook of Elemental Speciation. Handbook of Elemental Speciation II: Species in the Environment, Food, Medicine and Occupational Health.

[25] Shijo Y., Sato H., Uehara N., Aratake S. (1996). Simultaneous Determination of Trace Amounts of Copper, Nickel and Vanadium in Sea-water by High-performance Liquid Chromatography After Extraction and Back-extraction. Analyst.

[26] Agrawal Y.K., Menon S.K., Jain V.K. (2003). Liquid-liquid extraction, separation and preconcentration, membrane transportation and ICP-AES determination of vanadium with dibenzo-18-crown-6. Indian J. Chem..

[27] Filik H., Berker K.I., Balkis N., Apak R. (2004). Simultaneous preconcentration of vanadium(V/IV) species with palmitoyl quinolin-8-ol bonded to amberlite XAD 2 and their separate spectrophotometric determination with 4-(2-pyridylazo)-resorcinol using CDTA as masking agent. Anal. Chim. Acta.

[28] Seubert A. (2001). On-line coupling of ion chromatography with ICP-AES and ICP-MS. TrAC Trends. Anal. Chem..

[29] Gros N. (2013). Ion chromatographic analyses of sea waters, brines and related samples. Water.

[30] Singh R.P., Abbas N.M., Smesko S.A. (1996). Suppressed ion chromatographic analysis of anions in environmental waters containing high salt concentrations. J. Chromatogr. A.

[31] Lu H., Mou S., Riviello J.M. (2000). Effects of high concentration of chloride on the determination of trace anions by ion chromatography. J. Liq. Chromatogr. Relat. Technol..

[32] Wann C.C., Jiang S.J. (1997). Determination of vanadium species in water samples by liquid chromatography-inductively coupled plasma mass spectrometry. Anal. Chim. Acta.

[33] Aureli F., Ciardullo S., Pagano M., Raggi A., Cubadda F. (2008). Speciation of vanadium(iv) and (v) in mineral water by anion exchange liquid chromatography-inductively coupled plasma mass spectrometry after EDTA complexation. J. Anal. At. Spectrom..

[34] Chen Z.L., Owens G., Naidu R. (2007). Confirmation of vanadium complex formation using electrospray mass spectrometry and determination of vanadium speciation by sample stacking capillary electrophoresis. Anal. Chim. Acta.

[35] Lohmayer R., Reithmaier G.M.S., Bura-Nakić E., Planer-Friedrich B. (2015). Ion-Pair Chromatography Coupled to Inductively Coupled Plasma-Mass Spectrometry (IPC-ICP-MS) as a Method for Thiomolybdate Speciation in Natural Waters. Anal. Chem..

[36] Krachler M., Emons H. (2001). Urinary antimony speciation by HPLC-ICP-MS. J. Anal. At. Spectrom..

[37] Wallschläger D., London J. (2004). Determination of inorganic selenium species in rain and sea waters by anion exchange chromatography-hydride generation-inductively-coupled plasma-dynamic reaction cell-mass spectrometry (AEC-HG-ICP-DRC-MS). J. Anal. At. Spectrom..

[38] Novič M., Divjak B., Pihlar B., Hudnik V. (1996). Influence of the sample matrix composition on the accuracy of the ion chromatographic determination of anions. J. Chromatogr. A.

[39] Agatemor C., Beauchemin D. (2011). Matrix effects in inductively coupled plasma mass spectrometry: A review. Anal. Chim. Acta.

[40] Jen J.F., Yang S.M. (1994). Simultaneous speciation determination of vanadium(IV) and vanadium(V) as EDTA complexes by liquid chromatography with UV detection. Anal. Chim. Acta.

[41] Kanamori K., Sakurai M., Kinoshita T., Uyama T., Ueki T., Michibata H. (1999). Direct reduction from vanadium(V) to vanadium(IV) by NADPH in the presence of EDTA. A consideration of the reduction and accumulation of vanadium in the ascidian blood cells. J. Inorg. Biochem..

[42] Kanamori K., Kinebuchi Y., Michibata H. (1997). Reduction of vanadium(IV) to vanadium(III) by cysteine methyl ester in water in the presence of amino polycarboxylates. Chem. Lett..

[43] Kilibarda N., Afton S.E., Harrington J.M., Yan F., Levine K.E. (2013). Rapid speciation and determination of vanadium compounds using ion-pair reversed-phase ultra-high-performance liquid chromatography inductively coupled plasma-sector field mass spectrometry. J. Chromatogr. A.

[44] Mishra A.P., Khan R., Pandey R.R. (2009). Kinetic studies on effects of EDTA and surfactants on Reduction of vanadium(V) to vanadium(IV) in sulphuric acid medium. Indian J. Chem..

[45] Wang D., Sañudo-Wilhelmy S.A. (2008). Development of an analytical protocol for the determination of V (IV) and V (V) in seawater: Application to coastal environments. Mar. Chem..

[46] Baran E.J. (2000). Oxovanadium(IV) and oxovanadium(V) complexes relevant to biological systems. J. Inorg. Biochem..

[47] Reuter J.H., Perdue E.M. (1977). Importance of heavy metal-organic matter interactions in natural waters. Geochim. Cosmochim. Acta.

[48] Chen Z., Mahmudur Rahman M., Naidu R. (2007). Speciation of vanadium by anion-exchange chromatography with inductively coupled plasma mass spectrometry and confirmation of vanadium complex formation using electrospray mass spectrometry. J. Anal. At. Spectrom..

[49] LeGras C.A.A. (1993). Simultaneous determination of anions and divalent cations using ion chromatography with EDTA as eluent. Analyst.

[50] Legović T., Petricioli D., Ẑutić V. (1991). Hypoxia in a pristine stratified estuary (Krka, Adriatic Sea). Mar. Chem..

[51] Cukrov N., Doumandji N., Garnier C., Tucaković I., Dang D.H., Omanović D., Cukrov N. (2020). Anthropogenic mercury contamination in sediments of Krka River estuary (Croatia). Environ. Sci. Pollut. Res..

[52] Cukrov N., Frančišković-Bilinski S., Mikac N., Roje V. (2008). Natural and anthropogenic influences recorded in sediments from the Krka river estuary (Eastern Adriatic coast), evaluated by statistical methods. Fresenius Environ. Bull..

[53] Louis Y., Garnier C., Lenoble V., Mounier S., Cukrov N., Omanović D., Pižeta I. (2009). Kinetic and equilibrium studies of copper-dissolved organic matter complexation in water column of the stratified Krka River estuary (Croatia). Mar. Chem..

[54] Legović T. (1991). Exchange of water in a stratified estuary with an application to Krka (Adriatic Sea). Mar. Chem..

[55] Pađan J., Marcinek S., Cindrić A.M., Layglon N., Lenoble V., Salaün P., Garnier C., Omanović D. (2019). Improved voltammetric methodology for chromium redox speciation in estuarine waters. Anal. Chim. Acta.

[56] Pađan J., Marcinek S., Cindrić A.M., Layglon N., Garnier C., Salaün P., Cobelo-García A., Omanović D. (2020). Determination of sub-picomolar levels of platinum in the pristine Krka River estuary (Croatia) using improved voltammetric methodology. Environ. Chem..

[57] Marcinek S., Santinelli C., Cindrić A.-M., Evangelista V., Gonnelli M., Layglon N., Mounier S., Lenoble V., Omanović D. (2020). Dissolved organic matter dynamics in the pristine Krka River estuary (Croatia). Mar. Chem..

[58] Cukrov N., Cmuk P., Mlakar M., Omanović D. (2008). Spatial distribution of trace metals in the Krka River, Croatia: An example of the self-purification. Chemosphere.

[59] Grẑetić Z., Precali R., Degobbis D., Škrivanić A. (1991). Nutrient enrichment and phytoplankton response in an Adriatic karstic estuary. Mar. Chem..

