# Supplementary material for: Redox Speciation of Vanadium in Estuarine Waters Using Improved Methodology Based on Anion Exchange Chromatography Coupled to HR ICP-MS System
Source: Molecules. 2021 Apr 22;26(9):2436. doi: 10.3390/molecules26092436 (PMC8122592; doi:10.3390/molecules26092436)
Supplement: Supplementary file 1 [file molecules-26-02436-s001.zip › molecules-1155858-supplementary-1-done.pdf]

molecules

MDPI

Supplementary

Redox Speciation of Vanadium in Estuarine Waters Using Improved Methodology Based on Anion Exchange Ion Chromatography Coupled to HR ICP-MS System

Lucija Knežević [1], Dario Omanović [1], Niko Bačić [1], Jelena Mandić [2] and Elvira Bura Nakić [1] *

[1] Division for Marine and Environmental Research, Ruđer Bošković Institute, Bijenička cesta 54, 10000, Zagreb,
[2] Laboratory for Chemical Oceanography and Sedimentology of the Sea, Institute of oceanography and Fisher-ies, Šetalište Ivana Meštrovića 63, 21000, Split, Croatia
* Correspondence: ebnakic@irb.hr (E.B.N.)

Results and discussion

Analytical Validation of the Data

For the accurate V(V) concentration determination due to the high influence of sample matrix, 4-point standard addition method was employed. On Figure S1a. is shown example of chromatograms obtained using the stated method on one of the estuarine samples (SB, Jan.2020.) Samples were spiked using V(V) standard solution, with the exact spike concentration being 20 nmol L^{-1}. Concentration range studied was up to 80 nmol L^{-1}. Peak heights were used in the process of data treatment for obtaining quantitative information on V(V) concentration. In order to justify usage of peak heights as analytical signal in the process of V(V) concentration determination, on Figure S1b and S1c are shown standard additions of V(V) standard in the sample of SB Jan.2020 as a function of peak heights and peak areas of corresponding chromatograms, respectively. High linearity and obtained V(V) concentrations values in both presented cases further support high accuracy of proposed method.

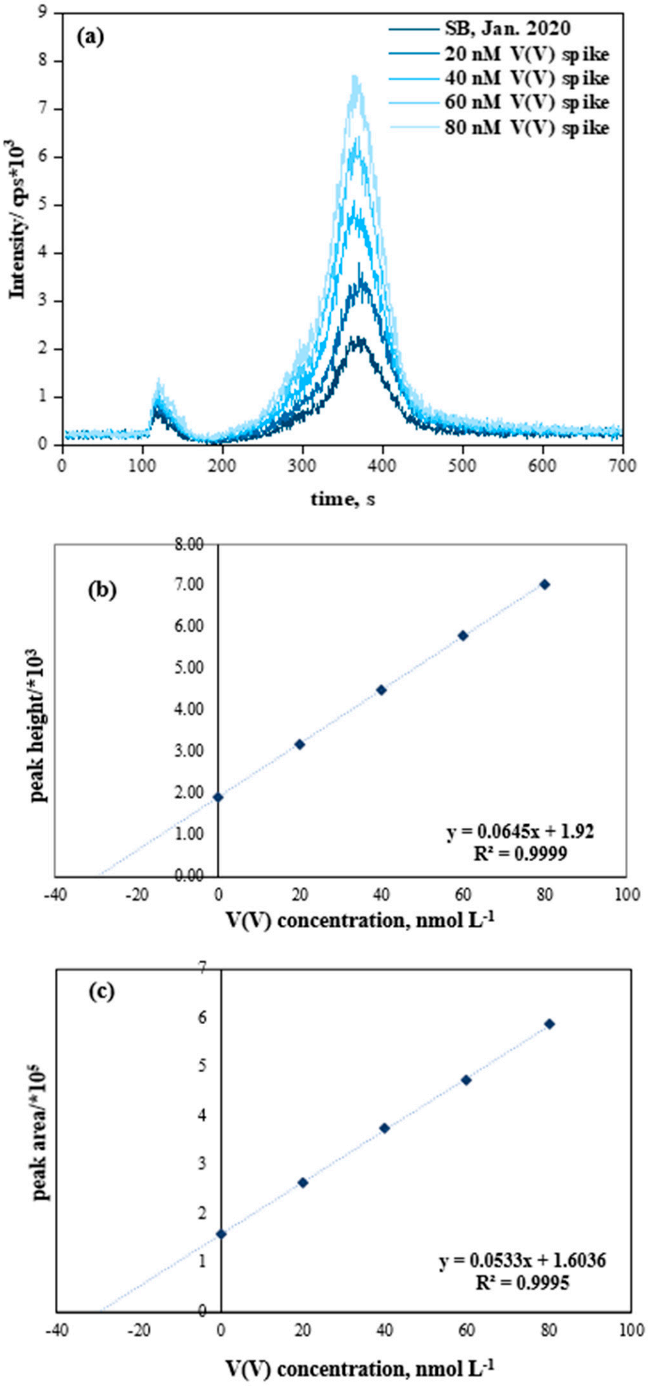

Figure S1. (**a**) Example of obtained chromatograms using standard addition method for V(V) concentration determination on the estuarine sample SB. Jan.2020. (S=35). (**b**) linear fitting of V(V) standard additions in the sample of SB. Jan. 2020. as a function of peak heights (calculated V(V) = 29.8 ± 1.79 nmol L^{-1}) (**c**) linear fitting of V(V) standard additions in the sample of SB.Jan.2020. as a function of peak areas (calculated V(V) = 30.1 ± 3.15 nmol L^{-1}).

The detection limits were determined using modified blank determination method. They were evaluated to be matrix dependant as well, with the exact values being 0.6 nmol L^{-1} in the freshwater and 2 nmol L^{-1} in the seawater. Limits of quantification were determined as 3 times of detection limits: 1.8 nmol L^{-1} in the freshwater and 6 nmol L^{-1} in the seawater. The reproducibility of the method was tested by triplicate analysis of seawater sample (Salinity=35). The confidence interval was 0.8% while relative standard deviation amounted to 1.1 % for V(V) species showing high accuracy. Repeatability accuracy of peak height for V(V) species was > 95 % (n=5). Recovery of V(V) species was evaluated by

spiking estuarine sample with known amount of V(V) standard. Results showed high recovery of V(V) species, with recovery values of 98% (with relative standard deviation of 2.6%, n=3) of V(V) standard. Performed stability tests of V(V) species obtained by spiking a seawater sample with known amount of V(V) standard (40 nmol L^{-1}) showed satisfactory results. In the Figure S2 are presented chromatograms supporting Figure 2d. in the main manuscript.

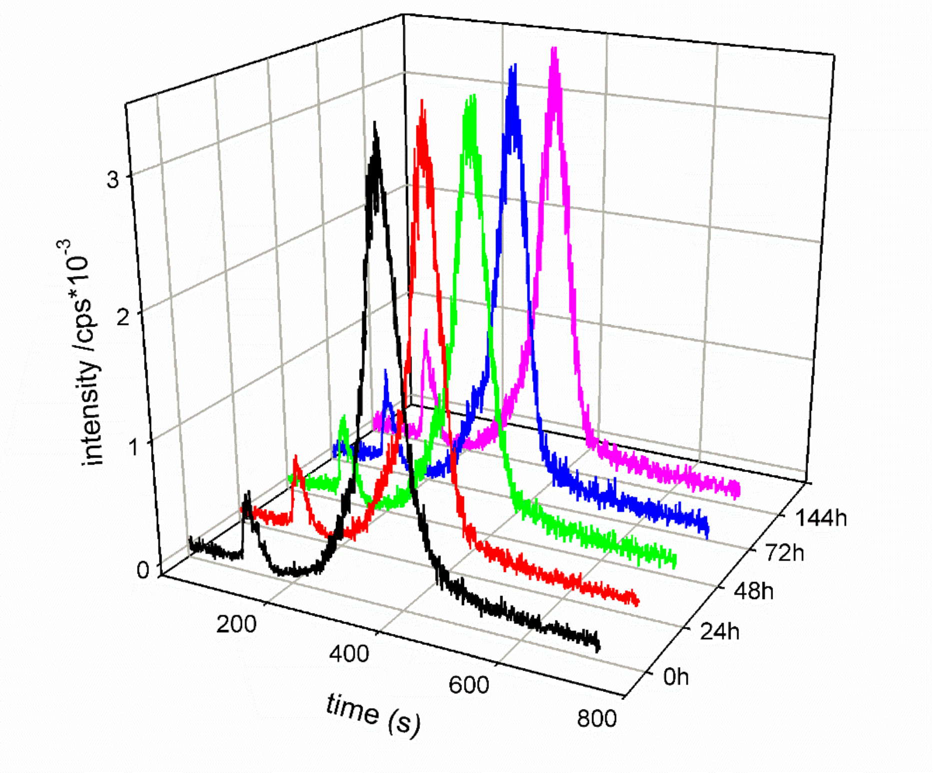

Figure S2. Chromatograms showing stability of V(V) in the seawater sample spiked with 40 nmol L^{-1} V(V) standard measured repeatively in the period of 144 hours.

All of the analytical figures of merit as well as additional informations on method are shown in Table S1 and compared with the work done by Li et al. (2018) as it served as a basis for the method presented in this manuscript. Also, comparison with the work done by Wann et al. (1997) is shown due to the similarity in the matrix of the processed samples.

Table S1. Comparison of the method information and analytical figures of merit of the presented work and work done by Li et al. [1] and Wann et al. [2].

**Method information and analytical merit**	**Li et al. 2018.**	**Wann et al. 1997.**	**p.w.**
Instrumentation	IC-ICP MS	RP LC-ICP MS	IC-ICP MS
Mobile phase composition	2 mmol L^{-1} EDTA, 80 mmol L^{-1} ammonium bicarbonate, 3% acetonitrile	3 mmol L^{-1} EDTA, 0.55 mmol L^{-1} tetrabutylammonium phosphate, 12% (v/v) methanol solution	8 mmol L^{-1} EDTA, 40 mmol L^{-1} ammonium bicarbonate, 40 mmol L^{-1} ammonium sulphate, 3% acetonitril
Samples matrix	Coke pore water samples, bacterial cultures	Seawater reference material	Estuarine samples (S =4–35)
Method of V(V) concentration determination	Calibration curves	Calibration curves	Standard addition method
Retention time V(V)-EDTA, min	4.9	3.221	5.0

Recovery	97 %	93–104 %	98 %
LOD	V(V) 19.63 nmol L^{-1}	V(V) 0.078 nmol L^{-1}	V(V) 0.6 nmol L^{-1} (in the freshwater)<br>2 nmol L^{-1} (in the seawater)
LOQ	-	-	V(V) 1.8 nmol L^{-1} (in the freshwater)<br>6 nmol L^{-1} (in the seawater)
RSD, %	0.92	-	1.1
Repeatability of peak height, %	-	5.6	4.6

*p.w. – present work.

Distribution of V redox speciation in the Krka River estuary samples

Table S1. Numerical values of total dissolved V, determined dissolved V(V) concentration and calculated percentage of V(IV) within the Krka River estuary samples.

Sample	Total Dissolved V Determined by ICP-MS / nmol L^{-1}	st. Deviation / nmol L^{-1}	Dissolved V(V) Determined by IC-ICP-MS / nmol L^{-1}	Replicate	st. Deviation / nmol L^{-1}	% V(IV)
SM – 0.5 m depth (January 2020)	18.4	1.2	18.2	1	3.6	1.08
SM – 0.9 m depth(January 2020)	25.3	0.4	24.2	2	0.5	4.35
SM –5 m depth(January 2020)	30.7	1.2	26.4	1	1.9	14.00
SM – 6.1 m depth(January 2020)	30.2	1.2	29.8	2	1.79	1.32
M – 0. 5 m depth (January 2020)	21.6	1.0	21.0	2	4.51	2.70
M – 2.2 m depth (January 2020)	20.7	0.6	19.0	1	4.6	8.21
LV – 1.8 m depth (January 2020)	24.5	1.4	21.8	2	0.1	11.00
LV – 2.8 m depth (January 2020)	25.9	1.2	23.1	2	1.66	10.81
LV – 7 m depth (January 2020)	33.8	0.4	26.3	2	1.22	22.18
LV – 10 m depth (January 2020)	37.0	0.8	31.1	1	3.34	15.94
SM – 0.4 m depth(November 2019)	12.3	0	12.1	1	1.98	1.63
SM – 1.5 m depth(November 2019)	20.1	0	19.4	1	3.62	3.48
SM – 3.5 m depth(November 2019)	29.94	0	26.3	1	0.37	12.16
SM – 5.0 m depth(November 2019)	24.06	0	17.8	1	1.27	26.01
SM – 6.0 m depth(November 2019)	21.65	0	17.1	1	0.14	21.01

Materials and methods

Table S2. Operating conditions of High Resolution Inductively Coupled Plasma Mass Spectrometry (HR ICP-MS).

Instrument	Element 2 (Thermo, Germany)
**RF power**	1200 W
**Cool (plasma) gas**	15.0 L min^{-1}
**Auxiliary gas**	0.92 L min^{-1}
**Sample gas**	1.00−1.02 L min^{-1}
**Nebulizer**	Sea-spray, 0.4 mL min^{-1}
**Spray chamber**	Twister, 50 mL, Cyclonic
**Sample cone**	Nickel
**Skimmer cone**	Nickel, „H“ model

References:

1. Li, X.S.; Le, X.C. Speciation of vanadium in oilsand coke and bacterial culture by high performance liquid chromatography inductively coupled plasma mass spectrometry. *Anal. Chim. Acta* **2007**, *602*, 17–22, doi:10.1016/j.aca.2007.09.004.
2. Wann, C.C.; Jiang, S.J. Determination of vanadium species in water samples by liquid chromatography-inductively coupled plasma mass spectrometry. *Anal. Chim. Acta* **1997**, *357*, 211–218, doi:10.1016/S0003-2670(97)00570-9.
